# Improving olfactory assessment: an item response theory analysis of the American English version of the Sniffin’ sticks identification subtest

**DOI:** 10.3389/fpsyg.2026.1661164

**Published:** 2026-01-29

**Authors:** Eva Tolomeo, Leognano Ceraudo, Ryann Kolb, Pamela H. Dalton, Marco Tullio Liuzza, Valentina Parma

**Affiliations:** 1 Department of Health Sciences, University “Magna Graecia”, Catanzaro, Italy; 2 Monell Chemical Senses Center, Philadelphia, PA, United States; 3 Department of Developmental Psychology and Socialization, University of Padova, Padova, Italy; 4 Department of Otorhinolaryngology – Head and Neck Surgery, University of Pennsylvania, Philadelphia, PA, United States

**Keywords:** differential item functioning, item response theory, odor identification, olfactory assessment, Sniffin’ sticks

## Abstract

**Introduction:**

The Sniffin’ Sticks Extended Test (SSET) is one of the most widely used tools for assessing olfactory function in research and clinical settings. Despite its broad application, a detailed psychometric evaluation of its items, including those within the identification subtest, remains limited. This study aimed to evaluate the reliability, validity, and item-level functioning of the SSET identification subtest using Item Response Theory (IRT), to identify potential weaknesses and propose possible areas for improvement.

**Methods:**

The study included 397 US-based participants (60.5% female; mean age 44.61 ± SD = 18.17 [45 ± 18]) who completed the American English version of the identification subtest of the SSET. IRT analyses were conducted using both a one-parameter (1PL) and a two-parameter (2PL) logistic model to estimate item difficulty and discrimination. A Differential Item Functioning (DIF) analysis was also performed to investigate potential sex-related biases in item responses.

**Results:**

Model comparison indicated that the 2PL model provided a better fit than the 1PL model. The 2PL analysis revealed that three items (i.e., *leather*, *turpentine*, and *pineapple*) exhibited low discrimination parameters, suggesting limited utility in distinguishing among different levels of olfactory ability. The DIF analysis found no evidence of differential item performance between male and female participants.

**Discussion:**

These findings support the use of IRT to identify poorly performing items, enabling the refinement of the SSET, to enhance its precision and reliability across populations. Future research should explore item revisions and extend psychometric evaluations to other subtests and samples.

## 1 Introduction

Olfactory function is increasingly recognized as a sensitive marker of neurological and psychiatric health. Impairments in odor perception, particularly in odor identification, are among the early detectable symptoms in a range of conditions, including Parkinson’s disease, and Alzheimer’s disease, and contribute to major depressive disorder, and schizophrenia (Doty, 2017; Rahayel et al., 2012; Wilson et al., 2009). As a result, reliable tools for assessing olfactory abilities have become informative in both research and clinical contexts.

One of the most widely used instruments to evaluate olfactory function is the Sniffin’ Sticks Extended Test (SSET; Hummel et al., 1997), a standardized battery comprising three subtests that assess complementary olfactory abilities: odor threshold, odor discrimination, and odor identification. These subtests are typically integrated into a global score - the TDI score (Threshold, Discrimination, Identification) - that has been validated across diverse languages and cultural contexts (Hummel et al., 2007; Kobal et al., 2000; Sorokowska et al., 2015). Among the three components, the odor identification subtest is particularly prominent in clinical practice due to its feasibility in routine screening, and it is often used in isolation (Quarmley et al., 2017; Dickmänken et al., 2024). The task involves 16 odorized felt-tip pens, each presented in a four-alternative forced-choice format. Participants are asked to sniff each pen and select the correct label from among four written options. This subtest is particularly sensitive to alterations in higher-order odor-related cognitive processes, including semantic memory, lexical retrieval, cultural familiarity with odors, and attentional and executive functions (Hedner et al., 2010).

Despite its extensive research and clinical use (Sorokowska et al., 2015), the internal structure of the identification test, and the psychometric functioning of its items have received poor attention, and item-level performance, such as poor discrimination, redundancy, or cultural bias, may hamper the interpretation of total scores. Although designed to include familiar stimuli, diverse cross-cultural validation studies (Konstantinidis et al., 2008; Kamrava et al., 2021; Sai-Guan et al., 2020) have found that some items, like *turpentine* or *licorice*, have shown low recognizability, semantic ambiguity, or limited cultural relevance, with some odors demonstrating low identification rates (<70%), leading researchers to replace or adapt them in local versions.

Traditional analyses based on Classical Test Theory (CTT) have critical limitations, especially in the context of olfactory testing. CTT assumes constant measurement error across ability levels, and its parameters (e.g., item-total correlations, Cronbach’s alpha) are sample-dependent, limiting their generalizability to new populations (Embretson and Reise, 2000). These assumptions are particularly problematic in the context of olfactory testing, where item difficulty and discrimination can vary as a function of cultural familiarity with odor stimuli, linguistic accessibility, and non-normal distributions of olfactory ability (e.g., ceiling effects in normosmic samples). In contrast, Item Response Theory (IRT) offers a more robust psychometric framework for evaluating the properties of individual items (Embretson and Reise, 2000). By modeling the probability of a correct response as a function of a participant’s latent trait (in this case, olfactory identification ability), IRT allows for the joint estimation of individuals’ latent abilities and item parameters on a common measurement scale, typically expressed in log-odds units (Carlson and Von Davier, 2017). More specifically, according to Embretson and Reise (2000), IRT addresses several limitations inherent in CTT by relaxing key classical assumptions and introducing alternative measurement principles, including the following: (i) measurement precision varies as a function of ability rather than being constant across scores; (ii) score interpretation is grounded in the relative location of individuals and items on a common latent continuum; (iii) comparisons are optimized when item difficulty is matched to individual ability; (iv) interval-level scaling is achieved through the measurement model itself rather than through distributional assumptions; and (v) under appropriate model fit and adequate coverage of the latent trait, item parameters are less dependent on strictly representative samples than CTT-based indices.

Within this framework, IRT estimates several item-level parameters, including: difficulty, or the level of ability at which there is a 50% chance of correctly identifying the odor; discrimination, which reflects how well an item distinguishes between individuals with different ability levels; and, in specific models, guessing, which reflects the probability that an examinee will answer the item correctly by random guessing alone, independently of the latent trait level (Embretson and Reise, 2000; Carlson and von Davier, 2017). In the context of olfactory testing, ability levels estimated by IRT represent a continuous latent trait reflecting olfactory function. Clinical categories such as anosmia, hyposmia, and normosmia correspond to ranges or thresholds along this continuum, allowing for discrete classification based on continuous ability estimates. Furthermore, for multiple-choice instruments such as the SSET, the nominal response model (Darrell Bock, 1972) within the IRT framework estimates the probability that a respondent will select each response category as a function of the latent trait, thus providing empirical evidence on whether each distractor works as intended or requires revision. Finally, IRT supports the analysis of Differential Item Functioning (DIF), which is crucial for identifying whether certain items perform differently across subgroups (e.g., males vs. females), despite equivalent underlying ability, thereby addressing issues of potential bias in test interpretation (Zumbo, 1999). For these reasons, in the present study we decided to adopt an IRT framework primarily for its broader advantages in the comprehensive psychometric evaluation and refinement of the Sniffin’ Sticks Identification Test.

Recent work by Tolomeo et al. (2024) has highlighted the need for a more comprehensive psychometric evaluation of olfactory tests, emphasizing the importance of complementing the use of aggregated score models with item-level models. For these reasons, the present study aims to address these gaps by performing a detailed IRT-based evaluation of the American English version of the SSET odor identification subtest. In this manuscript, we aim to: (i) estimate item difficulty and discrimination parameters of the American English version of the Sniffin’ Sticks Olfactory Identification (ID 16) subtest; (ii) examine measurement precision across the latent ability continuum, as estimated from responses in our sample; (iii) assess sex-related Differential Item Functioning; (iv) investigate the functioning of distractors in the OI items, evaluating the attractiveness of incorrect response options across varying levels of olfactory ability. Through this psychometric refinement, we seek to improve the construct validity of the SSET and to enhance its research and clinical diagnostic utility, particularly in settings where early detection of olfactory deficits may inform treatment planning or differential diagnosis.

## 2 Materials and methods

### 2.1 Participants

This study includes 397 participants who were recruited through convenience sampling at the Monell Chemical Senses Center in Philadelphia (PA, USA). Recruitment occurred via flyers posted in the area, by leveraging the recruitment database at the Monell Center, and by word-of-mouth. Participants were excluded from the study if they met one or more of the following exclusion criteria: (i) a history of neurological or psychiatric disorders, (ii) previous head injuries involving a loss of consciousness, (iii) the presence of upper respiratory tract conditions at the time of testing (such as allergic or infectious rhinitis, or sinus infections), (iv) current pregnancy, and (v) ongoing oncological therapies. Participants completed the identification subtest of the Sniffin’ Sticks Extended Test (SSET) in English. Of these, 18 individuals were excluded due to missing values in both demographic variables and subtest items. The final sample included 379 participants [226 females (59.6%)], with a mean age of 44.61 years (SD = 18.17; age range: 18–83 years). No other demographical data (e.g., ethnicity, educational background) are available.

### 2.2 Procedure

The study received ethical approval from the Institutional Review Board of the University of Pennsylvania (Protocol Number: 844425), and all procedures were conducted in accordance with the principles of the Declaration of Helsinki. Written informed consent was obtained from all participants prior to their involvement. Each participant was assessed individually and was instructed to refrain from eating or drinking anything other than water for at least 1 h before the testing session. The assessment was part of a larger study that took approximately 60 min and was conducted in a quiet, well-ventilated room.

*The Odor Identification subtest of the Sniffin’ Sticks Extended Test*—American English version.

The identification subtest of the Sniffin’ Sticks Extended Test (Hummel et al., 1997) is designed to assess the ability to recognize and label familiar odors at a suprathreshold level. It consists of 16 felt-tip pens, each infused with a different odorant commonly encountered in everyday life (e.g., *rose*, *lemon*, and *fish*). During the assessment, the examiner presents one scented pen at a time by placing it approximately 2 cm in front of the participant’s nose for a few seconds. For each item, the participant is asked to choose the correct odor from four multiple-choice options. The response is selected from a standardized list, and only one choice is correct. The total score of the identification subtest ranges from 0 to 16, corresponding to the number of correct identifications.

### 2.3 Data analysis

#### 2.3.1 Testing IRT assumptions

We conducted all analyses with R (Version 4.3.1; R Core Team, 2023) and Rstudio (R Studio Team). We used the *lavaan* package (Rosseel, 2012) for Confirmatory Factor Analysis (CFA), the *ltm* package (Rizopoulos, 2006) for IRT modeling, the *mirt* package (Chalmers, 2011) for additional IRT diagnostics, and the *mokken* package (van der Ark, 2007) for assessing monotonicity. Before fitting the IRT models, we tested fundamental assumptions: (i) unidimensionality, (ii) local independence, and (iii) monotonicity (Meijer and Tendeiro, 2018).

We conducted a CFA with a one-factor solution to test the unidimensionality. Given the dichotomous nature of items, we used the Diagonally Weighted Least Squares estimator (DWLS) (Muthén, 1984; Li, 2016). We assessed the fit of the unidimensional model using both the scaled Chi-square test (scaled χ ^2^) and the following fit indices: scaled Comparative Fit Index (scaled CFI), scaled Tucker-Lewis Index (scaled TLI), scaled Root Mean Square Error of Approximation-scaled (scaled RMSEA), and Standardized Root Mean Square Residual (SRMR). We used other model fit indexes due to the χ ^2^ test sample size sensitivity (Alavi et al., 2020). We used the cut-off criteria for fit indexes proposed by Hu and Bentler (1999) to interpret the goodness of the model fit. Values below 0.05 indicated a good fit for RMSEA and SRMR, while for CFI and TLI values above 0.90 were considered acceptable (Hu and Bentler, 1999). Nonetheless, robust estimators such as DWLS, tend to produce downwardly biased RMSEA estimates and upwardly biased TLI and CFI values (Xia and Yang, 2019). Consequently, we deemed it necessary to report the *u* index (Revelle and Condon, 2025), a combination measure of scale unidimensionality and scale homogeneity, using the *unidim* function from the *psych* package (Revelle, 2025). Values of *u* close to 1 indicate strong unidimensionality (Revelle and Condon, 2025).

We employed the Q3 statistic (Yen, 1984), which examines residual correlations between item pairs after accounting for the latent trait, to assess the local independence. Values of Q3 greater than |0.20| were considered indicative of local dependence (Chen and Thissen, 1997; pp. 265–289). Consequently, we computed the Q3 matrix, and we identified potential item pairs with residual correlations exceeding this threshold.

Finally, to assess monotonicity, we used the *check.monotonicity* function from the *mokken* package (van der Ark, 2007), which tests whether the probability of a correct response increases monotonically with increasing levels of the latent trait.

#### 2.3.2 IRT model fitting and comparison

We fitted and compared two IRT models: the one-parameter logistic (1PL) model and the two-parameter logistic (2PL) model. The 1PL model assumes equal discrimination parameters across all items, while the 2PL model allows discrimination parameters to vary across items. The 1PL model was fitted using the *rasch* function from the *ltm* package (Rizopoulos, 2006), which estimates item difficulty parameters while constraining discrimination parameters to be equal across items. For the 2PL model, the *ltm* function has been employed with the formula specification including a latent trait parameter (z1), allowing both difficulty and discrimination parameters to vary across items (Rizopoulos, 2006). To compare the fit of these nested models, we employed a likelihood ratio test using the *anova* function, which computes the difference in log-likelihoods between the models and evaluates its statistical significance. Additionally, we compared the Akaike Information Criterion (AIC) and Bayesian Information Criterion (BIC) values, with lower values indicating better model fit (Akaike, 1974; Schwarz, 1978). For the best-fitting model, we examined item parameter estimates, including difficulty and discrimination parameters, to evaluate the psychometric properties of individual items. Discrimination parameters indicate how well items differentiate between individuals with different levels of the latent trait, while difficulty parameters indicate the level of the latent trait at which individuals have a 50% probability of endorsing the item. We deliberately chose not to employ a three-parameter logistic (3PL) IRT model (i.e., incorporating the guessing parameter) due to its well-documented issues with identifiability and estimation stability (Noventa et al., 2024).

#### 2.3.3 Test response function and test information function

Using the parameters from the best-fitting model, we converted the latent ability values ( θ ) into their corresponding total scores on the identification subtest. This approach is referred to as the Test Response Function (TRF; Embretson and Reise, 2000), a mathematical function that establishes a relationship between the latent ability measured by the test and the corresponding estimated raw score on the scale (Embretson and Reise, 2000). Consequently, given that a raw score of 8 or lower corresponds to functional anosmia, a raw score between 9 and 11 indicates hyposmia and a raw score of 12 or higher reflects normosmia (Oleszkiewicz et al., 2019), we were able to associate the θ values with diagnostic labels (functional anosmia, hyposmia, normosmia). Finally, using a Test Information Function (TIF; Boone and Staver, 2020) implemented via the *testinfo* function in the *mirt* package (Chalmers, 2011), we analyzed at which θ level the test provides the most information.

#### 2.3.4 IRT distractor analysis: nominal response model

As a secondary analysis, we conducted a one-dimensional Nominal Response Model (NRM; Darrell Bock, 1972) using the *mirt* package (Chalmers, 2011) to assess the behavior of the distractors in each item. We chose the one-dimensional NRM due to the nature of the data: each item had four distinct, unordered nominal response categories (Darrell Bock, 1972). This approach is particularly suited for multiple-choice tests where distractors may provide information about the respondent’s ability level (Darrell Bock, 1972). The category parameters (ak and d values) were estimated for each stimulus and each response option. In order to make the model more stable and interpretable, we set the least chosen distractor as the reference category (ak0 = 0, d0 = 0) and we constrained the discrimination value of the correct category for each item (ak3 = 3). The ak parameters represent the slope parameters for each response category, while the d parameters represent the intercepts. These parameters collectively determine the probability of selecting each response option as a function of the latent trait ( θ ). We also reported the confidence intervals (CI 95%) of the parameters (ak and d). The information matrix has been estimated using the “sandwich” method (Chalmers, 2018). This method ensures greater numerical stability even for models with slight misfit and guarantees greater control over type I error (Falk and Monroe, 2018). Additionally, we generated trace plots for selected items to visualize the category response functions across the θ continuum. These plots provide a graphical representation of how the probability of choosing each response option changes as a function of the underlying.

We assessed model fit using the M2 statistic (Maydeu-Olivares and Joe, 2006), and the RMSEA, SRMSR, TLI, and CFI indexes. Finally, we evaluated item-level fit using the S- χ2 Statistic (Orlando and Thissen, 2000). The results were examined for each of the 16 olfactory stimuli. We chose this approach because it provides specific information about potential misfits at the item level. In addition, we employed the Bonferroni correction to the *p*-values associated with the S- χ ^2^ statistics to account for multiple comparisons.

#### 2.3.5 Differential item functioning (DIF) analysis

To assess item measurement invariance across sex, we conducted a DIF analysis using the Logistic Regression (LR) method (Zumbo, 1999). As stated by Zumbo (1999; p.12), “DIF occurs when examinees from different groups show differing probabilities of success on the item after matching on the underlying ability that the item is intended to measure.” For dichotomous items, LR accommodates for both uniform and non-uniform DIF within a unified framework (Swaminathan, 1994). Non-uniform DIF happens when lower-scoring participants are more likely to succeed in the first group, while higher-scoring participants are more likely to succeed in the other group (Zumbo, 1999). The analysis was performed using the *difLogistic* function from the *difR* package (Magis et al., 2010), which implements logistic regression-based procedures for identifying DIF items. The DIF analysis was configured to detect both uniform and non-uniform DIF by setting the type parameter to “both.” For the sex-based comparison, we designated “female” as the focal group. The analysis utilized total test scores as the matching criterion to control for ability level differences between groups, ensuring that DIF detection was not confounded by overall performance differences between males and females. Finally, to control for multiple comparisons across the 16 items examined, we applied the Bonferroni correction method to adjust the *p*-values.

## 3 Results

### 3.1 Testing the IRT assumption

The CFA showed acceptable model fit, as evidenced by the following indices: scaled χ ^2^(104) = 132.39, *p* = 0.032; scaled CFI = 0.973; scaled TLI = 0.968; scaled RMSEA = 0.027, 90% CI [0.009, 0.040]; and SRMR = 0.075. These results were further supported by a unidimensionality index (*u* = 0.70), indicating moderate scale unidimensionality and homogeneity (Revelle, 2025).

The Q3 statistic (Yen, 1984) yielded the following item pairs’ residual correlation values: M = −0.046, Mdn = −0.047, range [−0.20, 0.11]. Only one item pair, *rose*-*peppermint* (r = −0.21), met the residual correlation threshold of |0.2| proposed by Chen and Thiessen (1997, pp. 265–289). This suggests that local independence was only minimally violated, thus justifying the continued use of IRT modeling without substantial adjustments. Finally, no item violates the assumption of monotonicity.

### 3.2 IRT model fitting and comparison

The 1PL model showed the following fit indexes: log-likelihood = −3014.414; AIC = 6062.828; BIC = 6129.766. In this model, the α value (discrimination) is fixed to 0.98 across items, but β values (difficulty) vary across items. In the 2PL model (log-likelihood −2954.234; AIC = 5972.468; BIC = 6098.469), both α and β values vary across items (item parameters reported in Supplementary Table S1).

We compared the 1PL and the 2PL model and found that the 2PL model is more compatible with the data, as indicated by the AIC and BIC indices (Table 1).

**Table 1:** Model fit statistics comparison between 1PL and 2PL item response theory models for the identification subtest.

Model	AIC	BIC	Log-likelihood	LRT statistic	df	*p*-value
1PL	6062.83	6129.77	−3014.41	_	_	_
2PL	5972.47	6098.47	−2954.23	120.36	15	<0.001

1PL, one-parameter logistic; 2PL, two-parameter logistic; AIC, Akaike Information Criterion; BIC, Bayesian Information Criterion; LRT, Likelihood Ratio Test; df, Degree of Freedom.

In the 2PL model, the β values range from −2 (*garlic*) to 0.2 (*turpentine*), while α values range from 0.3 (*leather*) to 3.9 (*peppermint*). Lower α values mean lower discrimination power. For instance, for the item *leather*, given a α value of 0.31 (std.error = 0.1) and a β value of −0.82 (std.error = 0.5), the probability of a correct response is 40% when θ = − 2, 48% when θ = − 1, and 56% when θ = 0. This probability increases to 64% when it rises to 1, and to 71% when it rises to 2, reflecting the item’s weak discriminatory power due to its low discrimination parameter. Conversely, for the item *apple*, given a α value of 0.89 (std.error = 0.2) and a β value of 0.15 (std.error = 0.1), the probability of a correct response is 12.87% when θ = − 2, 26.43% when θ = − 1, 46% when θ = 0, 68.07% when θ = 1, and 83.82% when θ = 2, suggesting a higher item discriminatory power. Alongside *leather*, the items *turpentine* ( α = 0.45; std.error = 0.1) and *pineapple* ( α = 0.40; std.error = 0.1) showed discrimination parameters that are comparatively lower than those of the remaining items. This reduced discriminative power implies that these items contribute less efficiently to differentiating individuals across θs . Notably, the item *peppermint* has the highest α value (3.9). Nonetheless, this item has a very small discriminatory power since when θ = − 1. Indeed, given a α value of 3.9 and a β value of −1.81, the probability of a correct response is 32.36% when θ = − 2. This probability increases to 95.86% when θ rises to −1, and to 99.91% when θ rises to 0. The item peppermint is not only excessively easy but also fails to effectively discriminate between individuals with low to moderate latent ability levels and those with high latent ability. In addition, this item exhibits the largest standard error of the parameter (1.01) among all items, further indicating its poor psychometric properties.

**Figure 1:**
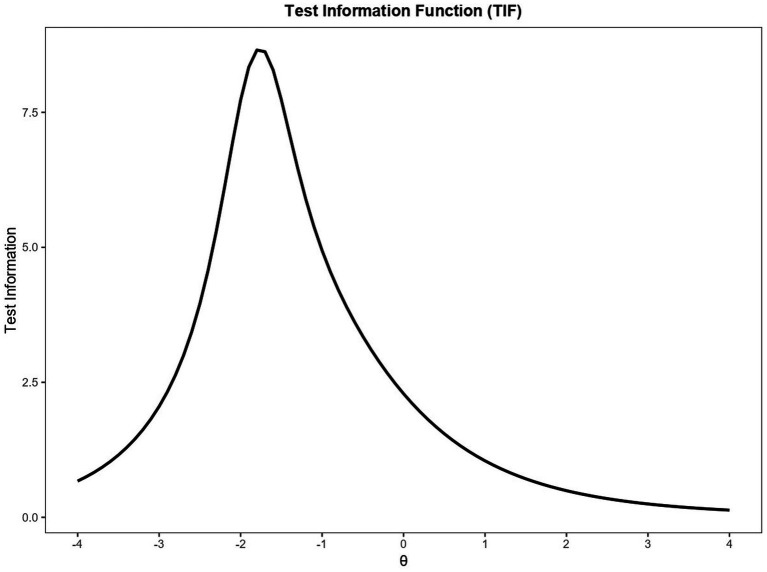
Identification subtest: two-parameter logistic model item characteristics curve.

We plotted the Item Characteristic Curves to better visualize the 2PL model item parameter (Figure 1).

### 3.3 IRT model fitting and comparison

For the TRF, using the α and β parameters from the 2PL IRT model, we estimated the corresponding total raw scores on the identification subtest across nine θ values (from −4 to 4). The most important results of this analysis suggest that: a θ value of −2 corresponds approximately to an expected score of 5, a θ value of −1 corresponds approximately to an expected score of 9, and a θ value of 0 corresponds approximately to an expected score of 12. Given that a total raw score of 8 or lower on the identification subtest indicates functional anosmia, a score between 9 and 11 indicates hyposmia, and a score of 12 or higher indicates normosmia, we can infer with reasonable precision that: θ values ≤ − 2 correspond to functional anosmia, θ = − 1 corresponds to hyposmia, and θ values ≥ 0 corresponds to normosmia.

**Figure 2:**
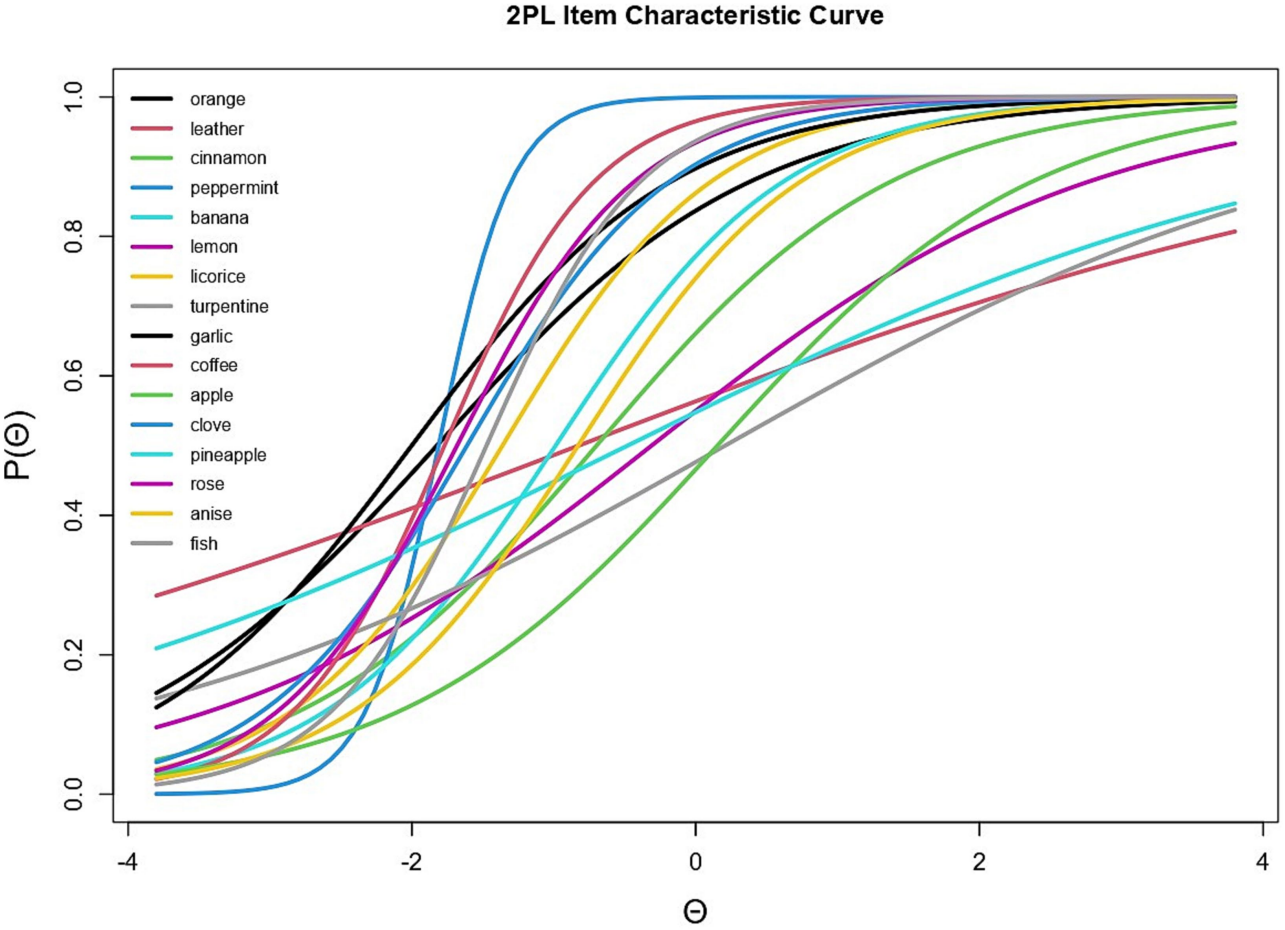
Identification subtest: test information function (TIF).

The TIF analysis suggested that the identification subtest shows greater measurement precision and information value within levels ranging from −2 to −1. As visible in Figure 2, this finding implies that the subtest provides optimal psychometric information precisely at the latent ability levels corresponding to functional anosmia and hyposmia. This psychometric property may enhance the clinical utility of the subtest.

### 3.4 IRT distractor analysis: nominal response model

As outlined below, the NRM analysis revealed adequate model fit across multiple indices. The M2 test statistic (54.14, df = 40; *p* = 0.067) suggested no severe misfit of the NRM to the data. The Root Mean Square Error of Approximation was 0.031, with a 90% confidence interval ranging from 0.000 to 0.050. This value falls well below the conventional threshold of 0.05 for acceptable fit and approaches the 0.03 criterion for excellent fit (Hu and Bentler, 1999). The Standardized Root Mean Square Residual (0.058) was below the recommended cutoff of 0.08, indicating an acceptable absolute fit (Hu and Bentler, 1999). The Comparative Fit Index (0.936) exceeded the conventional threshold of 0.90, suggesting a good model fit relative to a baseline model (Hu and Bentler, 1999). However, the Tucker-Lewis Index (0.858) fell slightly below the recommended 0.90 criterion (Hu and Bentler, 1999), indicating a mild model misfit.

The individual item fit analysis, after applying the Bonferroni adjustment, revealed that four items (e.g., *orange*; *licorice*; *turpentine*; *pineapple*), albeit non-significantly, showed higher S- χ ^2^ statistical values, revealing potential discrepancies between observed and model predicted response patterns and suggesting potential violations of the assumed item response function. The remaining 12 items demonstrated adequate fit with adjusted *p*-values of 1.00, indicating that their response patterns were consistent with model expectations (Table 2).

**Table 2:** Identification subtest nominal response model: individual item fit analysis.

Item	S-χ^2^	df	RMSEA. S-χ^2^	*p*-raw. S-χ^2^	*p*-adjusted
Orange	38.28	23	0.04	0.02	0.38
Leather	35.93	33	0.01	0.33	1.00
Cinnamon	31.30	28	0.01	0.30	1.00
Peppermint	6.68	3	0.05	0.08	1.00
Banana	15.43	25	0.00	0.93	1.00
Lemon	18.96	22	0.00	0.64	1.00
Licorice	42.78	25	0.04	0.01	0.24
Turpentine	48.67	32	0.03	0.03	0.48
Garlic	16.73	16	0.01	0.40	1.00
Coffee	15.83	13	0.02	0.25	1.00
Apple	32.55	26	0.02	0.17	1.00
Clove	26.49	21	0.02	0.18	1.00
Pineapple	52.88	35	0.03	0.02	0.43
Rose	21.97	13	0.04	0.05	0.89
Anise	30.74	25	0.02	0.19	1.00
Fish	18.18	11	0.04	0.07	1.00

Dff: Degrees of Freedom; RMSEA. S- χ ^2^: Root Mean Square Error of Approximation of S- χ ^2^ Statistic; p-raw. S- χ ^2^: S- χ ^2^ raw p-value; p-Adjusted: S- χ ^2^ Bonferroni adjusted *p*-value.

The NRM parameters for all 16 items are presented in Table 3. Category intercept parameters showed the expected ordering for most items, with ak0 (the least chosen distractor) set to 0, ak1 (moderately rare distractor) showing intermediate values, ak2 (most frequent distractor) displaying higher values, and ak3 (correct response) constrained to 3.

**Table 3:** Identification subtest nominal response model parameters.

Item	Distractor 1	Distractor 2	Distractor 3	Correct category
Item 1	Strawberry ak0 = 0 d0 = 0	Pineapple ak1 = 0.55 (−0.5–1.6) d1 = 1.22 (0.4–2.1)	Blackberry ak2 = 1.36 (0.4–2.4) d2 = 1.90 (1.1–2.7)	Orange ak3 = 3 d3 = 4.03 (3.3–4.8)
Item 2	Smoke ak0 = 0 d0 = 0	Glue ak1 = 1.92 (0.7–3.1) d1 = 0.59 (0.05–1.2)	Grass ak2 = 3.06 (2.1–4) d2 = 1.19 (0–6–1.8)	Leather ak3 = 3 d3 = 2.13 (1.6–2.6)
Item 3	Chocolate ak0 = 0 d0 = 0	Vanilla ak1 = 0.70 (−0.4–1.8) d1 = 1.86 (0.9–2.8)	Honey ak2 = 1.40 (0.3–2.6) d2 = 2.55 (1.6–3.5)	Cinnamon ak3 = 3 d3 = 3.69 (2.7–4.6)
Item 4	Onion ak0 = 0 d0 = 0	Chive ak1 = −2.16 (−4.5–0.1) d1 = −5.10 (−14–3.7)	Fir ak2 = 0.16 (−0.6–−1) d2 = 2.25 (0.11–4.4)	Peppermint ak3 = 3 d3 = 9.37 (5.8–13)
Item 5	Coconut ak0 = 0 d0 = 0	Walnut ak1 = 0.82 (−0.03–1.7) d1 = 0.94 (−0.3–2.2)	Cherry ak2 = 2.33 (1.7–3) d2 = 2.40 (1.2–3.6)	Banana ak3 = 3 d3 = 3.96 (2.8–5.1)
Item 6	Peach ak0 = 0 d0 = 0	Apple ak1 = −3.69 (−7.5–0.1) d1 = −2.13 (−4–−0.2)	Grapefruit ak2 = 2.64 (−2–3.4) d2 = 2.31 (1.5–3)	Lemon ak3 = 3 d3 = 2.75 (2–3.5)
Item 7	Cookies ak0 = 0 d0 = 0	Cherry ak1 = −0.29 (−2.17–1.6) d1 = 0.11 (−1.1–1.3)	Spearmint ak2 = 0.70 (−0.3–1.7) d2 = 1.17 (0.4–2)	Licorice ak3 = 3 d3 = 3.54 (2.8–4.2)
Item 8	Mustard ak0 = 0 d0 = 0	Rubber ak1 = −0.39 (−3.4–2.6) d1 = 0.46 (−0.1–0.1)	Menthol ak2 = 0.87 (−1.2–2.3) d2 = 1.70 (1.3–2.2)	Turpentine ak3 = 3 d3 = 1.99 (1.5–2.4)
Item 9	Carrot ak0 = 0 d0 = 0	Sauerkraut ak1 = 0.72 (−0.2–2.6) d1 = 1.06 (−0.2–2.3)	Onion ak2 = 1.51 (0.3–2.6) d2 = 3.01 (1.8–4.3)	Garlic ak3 = 3 d3 = 5.40 (4.2–6.5)
Item 10	Wine ak0 = 0 d0 = 0	Cigarette ak1 = 0.86 (−0.3–2.1) d1 = −0.11 (−0.5–2.7)	Smoke ak2 = 1.08 (−1.9–−1.5) d2 = 0.40 (−1.5–2.4)	Coffee ak3 = 3 d3 = 5.13 (3.7–6.6)
Item 11	Orange ak0 = 0 d0 = 0	Peach ak1 = 1.89 (1.3–2.5) d1 = 2.28 (1.3–3.2)	Melon ak2 = 2.02 (1.5–2.5) d2 = 2.90 (2–3.9)	Apple ak3 = 3 d3 = 3.27 (2.3–4.2)
Item 12	Mustard ak0 = 0 d0 = 0	Pepper ak1 = 0.61 (−0.5–1.7) d1 = 0.97 (−0.5–2.4)	Cinnamon ak2 = 0.98 (−0.4–2.3) d2 = 2.27 (0.8–3.7)	Clove ak3 = 3 d3 = 4.83 (3.4–6.2)
Item 13	Peach ak0 = 0 d0 = 0	Plum ak1 = 0.88 (−1.2–3) d1 = 0.68 (0.2–1.1)	Pear ak2 = 0.80 (− 1.4–3) d2 = 0.86 (0.4–1.3)	Pineapple ak3 = 3 d3 = 1.87 (1.5–2.3)
Item 14	Raspberry ak0 = 0 d0 = 0	Cherry ak1 = −1.40 (−3.9–1.1) d1 = −1.89 (−6.7–2.8)	Chamomile ak2 = 1.45 (0.6–2.3) d2 = 3.16 (1.7–4.6)	Rose ak3 = 3 d3 = 5.81 (4.3–7.3)
Item 15	Honey ak0 = 0 d0 = 0	Rum ak1 = 1.85 (1.1–2.6) d1 = 2.69 (1–4.3)	Fir ak2 = 1.92 (1.9–2.4) d2 = 3.21 (1.6–4.8)	Anise ak3 = 3 d3 = 4.77 (3.1–6.4)
Item 16	Ham ak0 = 0 d0 = 0	Cheese ak1 = −0.68 (−4.3–2.9) d1 = 0.24 (−1–1.4)	Bread ak2 = −4.39 (−10.7–2) d2 = −1.55 (−3.2–0.1)	Fish ak3 = 3 d3 = 3.60 (2.7–4.5)

The parameters ak and d represent slope and intercept terms, respectively; To ensure model stability, ak0 and d0 are constrained to 0, while ak3 is fixed at 3; For interpretability, the least chosen distractors are assigned as reference categories; The parameters ak1, ak2, and ak3 should be interpreted relative to ak0 (reference slope), and d1, d2, and d3 relative to d0 (reference intercept). 95% confidence intervals of all parameters are in brackets (except for constrained parameters).

Several items exhibited negative category intercept parameters. The item *peppermint* showed a notably negative ak1 parameter (−2.167; CI = −4.5–0.12), indicating this distractor option may be particularly unattractive across ability levels, namely, a person with hyposmia and a person with normosmia are both not likely to choose this distractor option. Similarly, the item lemon displayed a negative ak1 parameter (−3.696; CI = −7.5–0.13), suggesting this distractor is rarely selected across ability levels. The item *fish* presented a negative ak2 parameter (−4.392; CI = −10.7–1.9), indicating that the most frequent distractor option performs counterintuitively. Additional items with negative parameters included *licorice* (ak1 = − 0.292; CI = −2.2–1.6), *turpentine* (ak1 = − 0.395; CI = −3.4–2.6), *rose* (ak1 = −1.401; CI = −3.9–1.1), and *fish* (ak1 = −0.687; CI = −4.3–2.3).

**Figure 3:**
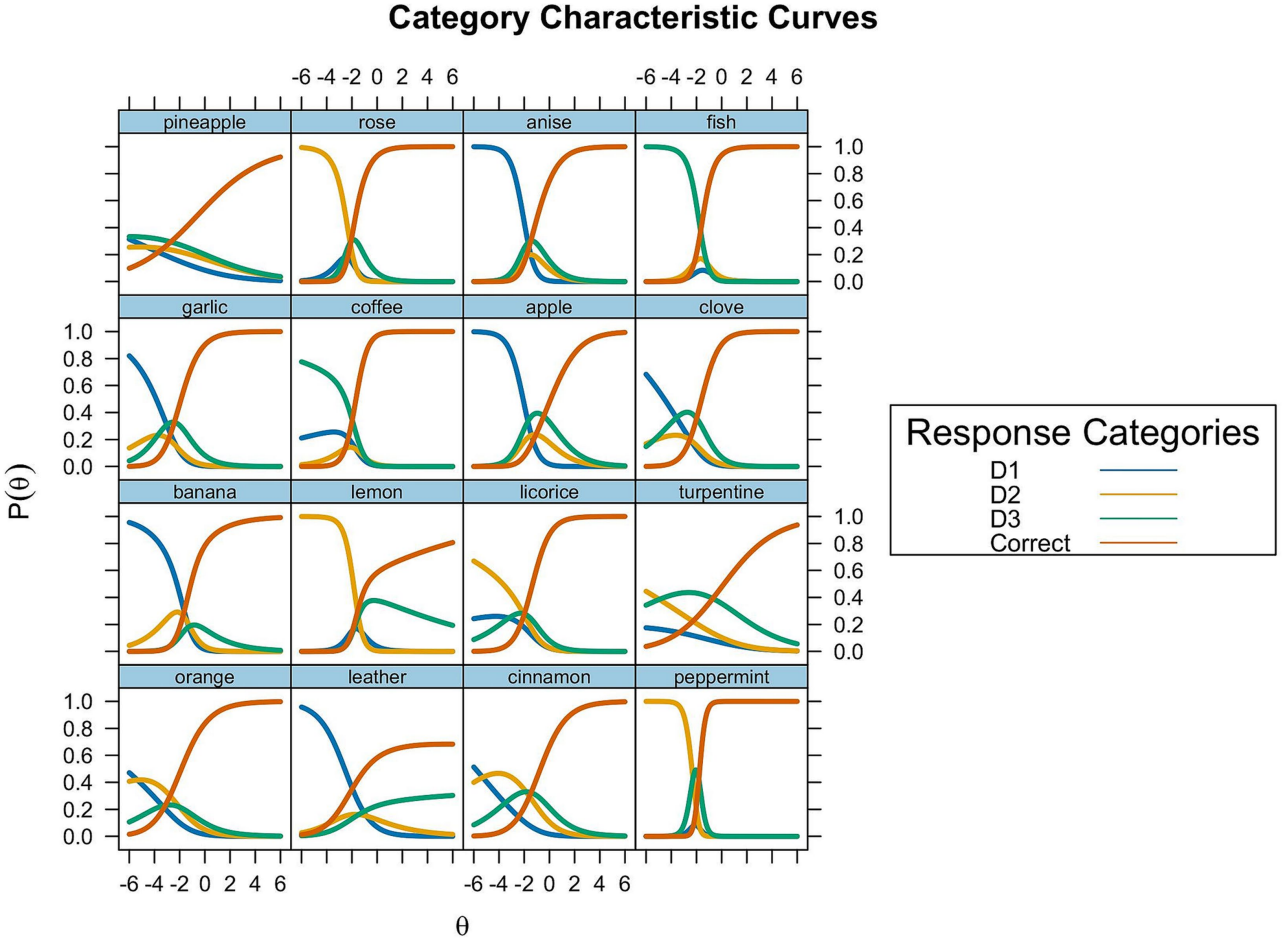
Identification items category characteristic curves. D1: The least chosen response category (blue lines); D2: Intermediate response category (yellow lines); D3: The most frequent response category (green lines); Correct: correct response category (red lines).

Lastly, the item *leather* shows an atypical behavior. The item parameters were: ak0 = 0, ak1 = 1.93 (CI = 0.72–3.1), ak2 = 3.06 (CI = 2.1–4), ak3 = 3, d0 = 0, d1 = 0.6 (CI = 0–1.2), d2 = 1.19 (CI = 0.6–1.8), d3 = 2.1 (CI = 1.6–2.6). If we calculate the probability of choosing a response category as θ varies, we can notice that both the correct answer and the most frequent distractor (*grass*) increase when θ increases. For example, when θ = 0, grass has a probability of 22.6%, when θ =1, grass has a probability of 27.7%, when θ =2, grass has a probability of 30.1%. These results suggest that normosmics are likely to be attracted by grass instead of *leather*. We reported the Category Characteristic Curves in Figure 3.

### 3.5 Differential item functioning (DIF) analysis

The Differential Item Functioning (DIF) analysis allowed us to assess whether certain items in the odor Identification subtest performed differently across sexes. After adjusting *p*-values using the Bonferroni correction, no items exhibited statistically significant DIF between males and females, as shown in Table 4. This finding aligns with prior research demonstrating that the Italian version of the SSET exhibits scalar measurement invariance across sexes (Tolomeo et al., 2024).

**Table 4:** Differential item functioning analysis results for identification subtest items by sex.

Item	*p*-adjusted	DIF
Orange	1.00	No
Leather	0.44	No
Cinnamon	1.00	No
Peppermint	1.00	No
Banana	1.00	No
Lemon	1.00	No
Licorice	1.00	No
Turpentine	1.00	No
Garlic	1.00	No
Coffee	1.00	No
Apple	1.00	No
Clove	1.00	No
Pineapple	1.00	No
Rose	1.00	No
Anise	0.30	No
Fish	1.00	No

*p*-adjusted, *p*-value adjusted with Bonferroni correction method; DIF, differential item functioning.

## 4 Discussion

This study aimed to evaluate the psychometric properties of the American English version of the Sniffin’ Sticks Identification subtest using the Item Response Theory. We analyzed how each item functions across the spectrum of olfactory ability (i.e., from anosmia to normosmia), gaining insights into both the difficulty and discriminatory capacity of the items. We further assessed the test’s measurement precision, investigated potential sex-related biases, and explored the performance of distractors across different levels of olfactory function. Our findings support the overall reliability and validity of the test, while also highlighting specific item-level issues that suggest the need for closer examination. These analyses aim to refine the interpretability and clinical utility of the Sniffin’ Sticks odor Identification subtest, particularly in contexts where early detection of olfactory dysfunction is crucial for diagnostic and treatment decisions.

Before applying the IRT models, we evaluated whether IRT core assumptions - unidimensionality, local independence, and monotonicity - were adequately met. This step was crucial to ensure the interpretability of the parameter estimates. Confirmatory Factor Analysis and the *u* index (Revelle, 2025), supported the unidimensional structure of the test. Local independence was only marginally violated for the items *peppermint* and *rose*, which exhibited a residual correlation approaching the threshold of *r* = −0.2 (Chen and Thissen, 1997; pp. 265–289). Finally, monotonicity was adequately respected.

These findings indicate that the English version of the SSET Identification subtest can be validly conceptualized as measuring a single latent ability (olfactory function), thus justifying the application of IRT to assess its item-level functioning (Meijer and Tendeiro, 2018). One-parameter IRT models assume that only difficulty varies across items, while two-parameter IRT models assume that both difficulty and discrimination vary across items (Meijer and Tendeiro, 2018). We deliberately chose to not employ a three-parameter logistic IRT model, given its well-documented issues with parameter identifiability and estimation stability (Noventa et al., 2024). Specifically, the guessing parameter (*c*-parameter) is particularly challenging to estimate reliably, especially for very easy items (Noventa et al., 2024). This concern is directly relevant to our data, as most items in the Identification subtest exhibited very high easiness, with items achieving correct response rates as high as 94% (e.g., *peppermint*). To better capture item functioning, we compared one-parameter and two-parameter IRT models. The 2PL model provided a superior fit, supporting the idea that items vary in their capacity to discriminate across ability levels. Despite the overall good model fit, specific items showed low discrimination or atypical response patterns. Specifically, the items *peppermint* and *leather* emerged as psychometrically problematic.

The item *peppermint*, despite having the highest discrimination value ( α = 3.9), failed to effectively distinguish between hyposmics versus normosmics, because its discriminatory power is concentrated at the lower end of the ability continuum. This makes it particularly useful for distinguishing anosmics from hyposmics but limits its effectiveness in differentiating individuals with moderate to high olfactory ability. This paradoxical result indicates that this item was excessively easy, meaning that even hyposmics correctly identified it, resulting in a ceiling effect. One plausible explanation is that *peppermint* might be a highly salient and trigeminal stimulating odor frequently encountered in daily life (e.g., toothpaste, chewing gum, herbal medicine). Its multisensory nature likely enhances recognition regardless of true olfactory ability, limiting its value in distinguishing across the upper-medium ability range. In contrast, the item *leather* showed low discrimination and would not help distinguish people with functional anosmia from people with hyposmia. Despite having relatively better discrimination parameters, both the *turpentine* and *pineapple* items still demonstrate suboptimal discrimination across the full range of levels, suggesting that these items may not adequately capture the intended psychometric construct.

To further explore these anomalies, particularly the atypical response patterns, we examined distractor functioning using the Nominal Response Model (NRM) (Darrell Bock, 1972). This model allowed us to assess how the probability of selecting each response option, including incorrect alternatives, varies as a function of olfactory ability. The NRM is particularly useful in multiple-choice tests, as it allows us to explore whether distractors provide meaningful information or introduce bias. Overall, model fit indices confirmed that the model appropriately represents the response data, and most distractors followed expected patterns, with low-ability individuals more likely to select unrelated options and higher-ability individuals choosing semantically or perceptually plausible alternatives. However, a subset of distractors showed either extremely low selection rates or unexpected behavior. In detail, the item *leather* was problematic since both the correct answer and the distractor grass were increasingly selected as ability increased, suggesting that the latter may act as a “false attractor” for high-ability individuals. Notably, this effect did not occur uniformly across all items, despite all following the same structure - one correct answer, one perceptually or semantically similar distractor, and two unrelated alternatives. This suggests that the response bias observed in this item stems not from the item format itself, but from a convergence of additional factors related to the specific properties of the item. As an example, the target odor *leather* may be relatively ambiguous or less prototypical, while grass represents a semantically salient, easily accessible alternative. The two options may also share overlapping perceptual features (e.g., earthy or vegetal notes), increasing the plausibility of the incorrect response. For individuals with higher olfactory sensitivity, such ambiguity may trigger a form of cognitive overinference, in which subtle perceptual cues are overanalyzed or mapped onto a more familiar conceptual label. This interaction between perceptual ability, stimulus ambiguity, and semantic salience may lead to a paradoxical decrease in accuracy among individuals with high ability - an effect consistent with expertise-related biases in other perceptual domains. This supports the interpretation that stimulus ambiguity and semantic overlap can introduce confusion even in those with high perceptual skills. These findings highlight the importance of considering not only correct response rates but also the psychometric behavior of distractors when evaluating item functioning.

Furthermore, analysis of the TIF, which identifies the θ levels at which the test provides optimal precision and measurement information (Boone and Staver, 2020), revealed that the identification subtest is most informative for θ values ranging between −2 and −1. As indicated by the Test Response Function analysis, these levels correspond to the clinical classifications of anosmia ( θ ≤ − 2) and hyposmia ( θ = − 1). These findings underscore the clinical utility of this subtest for assessing olfactory impairment severity.

Finally, we performed a Differential Item Functioning (DIF) analysis to evaluate whether any items perform differently across sexes, which is critical to ensure the test’s validity (Zumbo, 1999). Using logistic regression methods, we assessed both uniform and non-uniform DIF, matching participants on overall ability to control for group differences in olfactory function. The analysis was corrected for multiple comparisons to reduce false positives. Our results indicated no evidence of DIF across sexes, suggesting that the items measure olfactory ability equivalently in males and females, thus supporting the subtest’s measurement invariance and reinforcing its applicability across groups.

Taken together, these analyses deepen our understanding of the Sniffin’ Sticks Identification subtest by confirming its measurement invariance across sexes and providing detailed insights into the role of distractors in shaping item responses. Recognizing that certain distractors may disproportionately attract individuals at specific ability levels can inform targeted revisions to items, enhancing the test’s precision and clinical utility. These findings underscore the importance of examining not only the correct responses but also the patterns of incorrect answers when refining olfactory assessment tools. Given their problematic behavior, these items should be considered for revision to enhance the overall validity and precision of the subtest, particularly at the low and moderate ends of the ability continuum.

Beyond these psychometric considerations, our findings have broader implications for the maintenance of olfactory tests over time. Odor identification is not merely a perceptual task but also relies on higher-order cognitive processes such as semantic memory, language access, and cultural familiarity, as consistently documented in the literature (Cain et al., 1998; Huisman and Majid, 2018). These well-established factors provide an important theoretical framework for interpreting differences in item performance and may help explain why certain odor stimuli exhibit lower discrimination or greater susceptibility to cultural variation. These dimensions can shift over time due to changes in exposure, product availability, or cultural practices. Test validity is therefore not a static property. An item that performs well in one temporal or cultural context may fail to do so in another. For this reason, we strongly suggest routine psychometric reassessment and periodic item calibration. Future longitudinal studies will determine the cadence of reassessment. Such practices are standard in large-scale educational testing and should be considered best practice in sensory and clinical assessments as well (American Educational Research Association et al., 2014). Regular test updates would ensure that measurement remains both accurate and valid, and that test performance reflects the targeted construct rather than cultural familiarity or other factors.

This study has some limitations. First, we used a convenience sample, which mainly consisted of healthy individuals, and this may limit the generalizability of the findings to clinical populations with olfactory dysfunction. Future studies should assess the item’s functioning across diverse diagnostic groups to enhance external validity. Second, the analysis was cross-sectional, and this limits the possibility of drawing conclusions about the temporal stability or test–retest reliability of the item parameters. Third, this study is based exclusively on the US version of the Sniffin’ Sticks identification subtest. Although the application of IRT allowed for a detailed examination of item-level psychometric properties within this version, the findings may not be fully generalizable to other cultural or linguistic adaptations of the test. Odor identification performance is known to be influenced by cultural familiarity, semantic labeling, and prior exposure to specific odorants, which may affect item difficulty and discrimination parameters across populations. Consequently, the assumption of cultural invariance of the items cannot be guaranteed. Items that function adequately in the U.S. version may exhibit differential item functioning in other cultural contexts, potentially limiting the cross-cultural applicability of the present results. Future studies should explicitly examine cultural invariance across different language versions and cultural groups to determine whether the IRT parameters identified here are stable across populations or require culture-specific calibration. Finally, although odor identification performance is known to be influenced by cognitive factors, such as semantic memory and language access, the present study did not include independent measures of these constructs. Consequently, convergent validity with related cognitive domains was not directly assessed. This reflects the primary aim of the study, which focused on the item-level psychometric evaluation of the Sniffin’ Sticks Identification Test using an Item Response Theory framework, rather than on the cognitive mechanisms underlying odor identification. Future studies integrating IRT-based analyses with external measures of semantic and linguistic processing may further clarify how these well-established factors contribute to item functioning and support a more comprehensive validity framework.

## 5 Conclusion

This study provides a comprehensive psychometric evaluation of the English version of the Sniffin’ Sticks Identification subtest using Item Response Theory, offering detailed insights into item functioning, test precision, and measurement validity. Our findings confirm that the test reliably assesses olfactory ability and performs consistently across sexes. However, variability in item-level parameters, particularly in terms of discrimination and distractor behavior, highlights areas for refinement. Overall, this work reinforces the value of IRT-based methods in sensory testing and highlights the importance of item-level analyses in improving the precision and interpretability of clinical olfactory tools.

## Data Availability

The datasets presented in this study can be found in online repositories. The names of the repository/repositories and accession number(s) can be found at: https://osf.io/3hjmw/?view_only=f93d1dd6812549b08d5503a207c4e49d.
