# Supplementary material for: Improving olfactory assessment: an item response theory analysis of the American English version of the Sniffin’ sticks identification subtest
Source: Front Psychol. 2026 Jan 29;17:1661164. doi: 10.3389/fpsyg.2026.1661164 (PMC12863060; doi:10.3389/fpsyg.2026.1661164)
Supplement: Supplementary file 1 [file Table_1.docx]

***Supplementary Material***

# Supplementary Data

### Table S1. Identification subtest Two-Parameters Logistic Model Parameters

####

| **Item** | $\beta$ | $\beta$  **Std. Error** | **z** | $\alpha$ | $\alpha$  **Std. Error** | **z** |
| --- | --- | --- | --- | --- | --- | --- |
| orange | -1.82 | 0.33 | -5.53 | 0.89 | 0.18 | 4.89 |
| leather | -0.82 | 0.48 | -1.71 | 0.30 | 0.13 | 2.35 |
| cinnamon | -0.69 | 0.16 | -4.18 | 0.95 | 0.18 | 5.27 |
| peppermint | -1.81 | 0.14 | -12.40 | 3.87 | 1.01 | 3.82 |
| banana | -0.98 | 0.16 | -5.98 | 1.23 | 0.21 | 5.75 |
| lemon | -0.31 | 0.18 | -1.65 | 0.64 | 0.15 | 4.20 |
| licorice | -1.36 | 0.19 | -7.13 | 1.34 | 0.23 | 5.83 |
| turpentine | 0.20 | 0.24 | 0.85 | 0.45 | 0.14 | 3.23 |
| garlic | -2.00 | 0.31 | -6.33 | 1.08 | 0.21 | 5.14 |
| coffee | -1.77 | 0.19 | -8.98 | 1.87 | 0.33 | 5.60 |
| apple | 0.15 | 0.13 | 1.12 | 0.89 | 0.18 | 4.96 |
| clove | -1.61 | 0.21 | -7.49 | 1.38 | 0.24 | 5.73 |
| pineapple | -0.47 | 0.30 | -1.55 | 0.40 | 0.13 | 2.96 |
| rose | -1.68 | 0.20 | -8.18 | 1.58 | 0.27 | 5.78 |
| anise | -0.82 | 0.14 | -5.56 | 1.26 | 0.21 | 5.75 |
| fish | -1.47 | 0.17 | -8.66 | 1.82 | 0.31 | 5.84 |

*Note.* $a$ *: discrimination parameters;* $\beta$*: difficulty parameters;* $\alpha$ *std.errors : estimation uncertainty of discrimination parameters;* $\beta$ *std.errors: estimation uncertainty of difficulty parameters; z: zeta-scores for discrimination and difficulty parameters*
